# D-Dimer as an Early Marker of Severity in Patients With Acute Superior Mesenteric Venous Thrombosis

**DOI:** 10.1097/MD.0000000000000270

**Published:** 2014-12-02

**Authors:** Shuofei Yang, Xinxin Fan, Weiwei Ding, Baochen Liu, Jiaxiang Meng, Kai Wang, Xingjiang Wu, Jieshou Li

**Affiliations:** From the Department of General Surgery, Jinling Hospital, Medical School of Nanjing University, Nanjing 210002, Jiangsu Province, P.R. China

## Abstract

No early serum marker of disease severity contributes to the treatment decision-making process of acute superior mesenteric venous thrombosis (ASMVT). This study aims to assess the value of serum D-dimer level in the first 3 days after admission as a severity marker of ASMVT patients.

From May 2010 to June 2014, 50 consecutive patients of ASMVT were enrolled in this observational study. The serum D-dimer level was measured on a daily basis during the first 3 days after admission as well as other laboratory-testing parameters, clinical score, and outcome variables recorded during the same period. The maximum and mean D-dimer values were analyzed and compared with other potential markers for prediction of multiple-organ dysfunction syndrome (MODS) and short-bowel syndrome (SBS). The correlation of D-dimer level with other potential severity markers and inflammation parameters were also studied.

Both maximum and mean D-dimer level during the first 3 days of admission were significantly higher in patients with several clinical variables such as death within 30 days, bowel resection, sepsis, abdominal compartment syndrome, MODS, and SBS. In addition, serum D-dimer level showed precise prediction for MODS and SBS, greater than l-lactate and intestinal-type fatty acid-binding protein (I-FABP). The D-dimer level was correlated well with l-lactate, I-FABP, and APACHE II score on the first 3 days of admission. Poor correlation of D-dimer level and inflammation parameters, white blood cell count, and C-reactive protein level, was detected.

D-dimer level could be an effective, early, and specific serum marker indicating the clinical evolution and outcome of ASMVT.

## INTRODUCTION

Acute mesenteric ischemia (AMI) is a devastating vascular emergency characterized by the “clinical triad” of pain out of proportion to physical findings, rapid and often forceful bowel evacuation, and a possible cause of embolus or thrombus, with a daunting mortality of 60% to 80%.^1,2^ As the least form of AMI, acute superior mesenteric venous thrombosis (ASMVT) accounts for approximately 6% to 9% of all cases and 1/1000 emergency department admissions.^3^ Once intestinal infarction occurs from venous engorgement and impeded arterial flow, there is high risk of extensive bowel resection due to few options to relieve the venous congestion. Successful outcome of ASMVT depends on prompt recognition and recanalization by immediate anticoagulation, endovascular therapy, or urgent surgery.^4,5^ Multiple-organ dysfunction syndrome (MODS) and short-bowel syndrome (SBS) induced by massive intestinal bacterial translocation and extensive transmural necrosis are the most lethal complications at early and later phase of ASMVT.^6^

Recent widespread use of computed tomography (CT) portography has facilitated early diagnosis of ASMVT with a sensitivity over 90%, but limited value in severity prediction.^7^ The optimal therapeutic management (type and duration of anticoagulants, thrombolysis, endovascular manipulations, or prompt surgery) are still controversial. It is of great significance to identify an early and easy serum marker to assist the treatment determination and prognostic evaluation.

The fibrinolytic marker D-dimer increases and accumulates as a result of local activation of intravascular coagulation at onset of AMI.^8^ D-dimer has been a validated prediction tool to assist clinical decision-making along the therapeutic management path for venous thromboembolism.^9,10^ Moreover, it was also reported as a sensitive marker for early detection of AMI and severity predictor of abdonimal crisis (eg, acute pancreatitis, strangulated intestinal hernia, abdominal aortic aneurysm).^11–18^ Elevated D-dimer level is a confirmed risk factor of mesenteric venous thrombosis (MVT) as well.^19,20^

In this observational pilot study, we aimed to identify the association between serum D-dimer level in the first 3 days of admission and several variables of clinical severity and outcome of ASMVT. Furthermore, we investigated whether it contributes to the clinical decision-making process by evaluating the accuracy of D-dimer level for prediction of MODS and SBS, and compare it with 2 other potential markers, intestinal-type fatty acid-binding protein (I-FABP) and l-lactate. The correlation of D-dimer level and other potential markers, clinical severity score, and inflammatory parameters was also studied.

## PATIENTS AND METHODS

### Study Design and Patients Selection

This study was approved by an institutional review board and ethic committee of Jinling Hospital. Written informed consent for enrolment into this study was given by all patients. In the present study, all consecutive adult patients (age ≥18 and ≤80 years) with ASMVT (within 1 week from the symptom onset) hospitalized in the Intestinal Stroke Center, Institute of General Surgery, Jinling Hospital, between May 2010 to June 2014 were considered eligible. The inclusion criteria were as follows: abdominal pain or distention, at least 1 mesenteric or portal vein occlusion on CT, digital subtraction angiography portography or confirmed during laparotomy exploration, intestinal wall injury at CT scan, alimentary intolerance requiring total parenteral nutrition (TPN). Patients with irreversible acute hepatic or renal failure, advanced malignancies, recurrent thrombosis, isolated portal venous thrombosis, known history of coagulative disorders or a recent history of myocardial infarction or cerebral infarction, surgical intervention before admission, transferring to other hospital within 3 days were excluded from the study. All patients received standard medical therapy on admission, then followed the algorithm of recanalization at our center.^21^

### Data Collection

Demographic information including age, sex, disease or family history, main symptoms and signs were assessed on admission. Charlson comorbidity index of total comorbidity burden and acute physiology and chronic health evaluation (APACHE) II score were calculated. The definitions of organ dysfunction were based on a score of ≥2 in the sequential organ failure assessment (SOFA) scoring system, and MODS was defined as the combined dysfunction of 2 major organ systems.^22^ SBS was diagnosed according to the definition in American Gastroenterological Association Statement.^23^ Etiology was determined by thrombophilia screen for protein C/S deficiency, antithrombin III deficiency, Factor V Leiden syndrome, prothrombin gene mutation, antiphospholipid or anticardiolipin syndrome, and dysfibrinogenemia.

Both the SOFA score and APACHE II score were assessed on a daily basis during first 3 days after admission. The development of other systemic and local complications, such as sepsis, hemodynamic unstability requiring vasoactive drugs, acute respiratory distress syndrome (ARDS) with mechanical ventilation, acute kidney injury (AKI) requiring hemodialysis, abdominal compartment syndrome (ACS), overall cost and duration of hospitalization, and the need for endovascular therapy or surgical intervention were also recorded into the electronic medical database.

In all patients, the serum D-dimer level was determined on admission and the next 2 days. It was tested by the particle-enhanced immunoturbidimetric assay Innovance D-DIMER (Siemens Medical Solutions, Malvern, PA) on the Behring Coagulation System analyzer, with the upper reference value of 550 μg/L. Maximum and mean D-dimer was defined as the highest and average level of all measurements in the first 3 days. Other routine serum parameters [eg, red blood cell count, white blood cell count (WCC), and platelet count, hemoglobin, hematocrit, hepatic and renal function markers, electrolyte, C-reactive protein (CRP), international normalized ratio, prothrombin time, activated partial thromboplastin time, fibrinogen, antithrombin III] and 2 other potential markers, l-lactate and I-FABP, were tested at the same time-point as the samples for D-dimer measurement.

### Statistical Analysis

Continuous variables were defined as means ± standard deviation if they were normally distributed, otherwise, medians (interquartile ranges) were represented. Categoric variables were described in absolute numbers and percentages. The Mann–Whitney *U*-test was used to explore the difference of serum D-dimer levels. To establish the optimal cut-off point of all potential markers, receiver operating characteristics (ROC) curves were drawn by plotting sensitivity against 1-specificity for all thresholds. Overall accuracy of the markers was summarized using area under the curve (AUC). The best cut-off point was defined as the maximum sum of sensitivity and specificity. Cut-off points were used to calculate sensitivity (Sn), specificity (Sp), positive and negative likelihood ratios (+LR and −LR), and positive and negative predict value (PP and NP). Univariate logistic regression analysis was performed to test the association of several potential markers and SBS, MODS, respectively. The agreement of the results between D-dimer level and other potential parameters were evaluated by Bland–Altman plots (Bland and Altman, 1986). The corresponding means, standard deviations, and binomial 95% confidence interval (95% CI) were calculated. The Spearman test was used to analyze correlations between serum D-dimer level and APACHE II score, I-FABP, CRP, WCC, and l-lactate variables. All statistical tests were 2-tailed, and the significance level was set at *P* < 0.05. Data were analyzed using SPSS version 18.0 for Windows (SPSS, Chicago, IL).

## RESULTS

### Demographic Information and Clinical Outcomes

A total of 50 patients [age of 46 (36–57.25) years, 29 males, 21 females] were enrolled in this study as scheduled. The demographic and clinical data were shown in Table 1. Thrombophilia was identified in 10 patients, protein C deficiency (n = 4), protein S deficiency (n = 3), antithrombin III deficiency (n = 2), factor V Leiden syndrome (n = 1), and antiphospholipid syndrome (n = 2). Among the 38 secondary ASMVT patients, liver cirrhosis with portal hypertension (n = 12) was the most common cause. On admission, patients have Charlson Comorbidity Index of 4.5 (3–6) and APACHE II score of 17.5 (13–21).

**TABLE 1:**
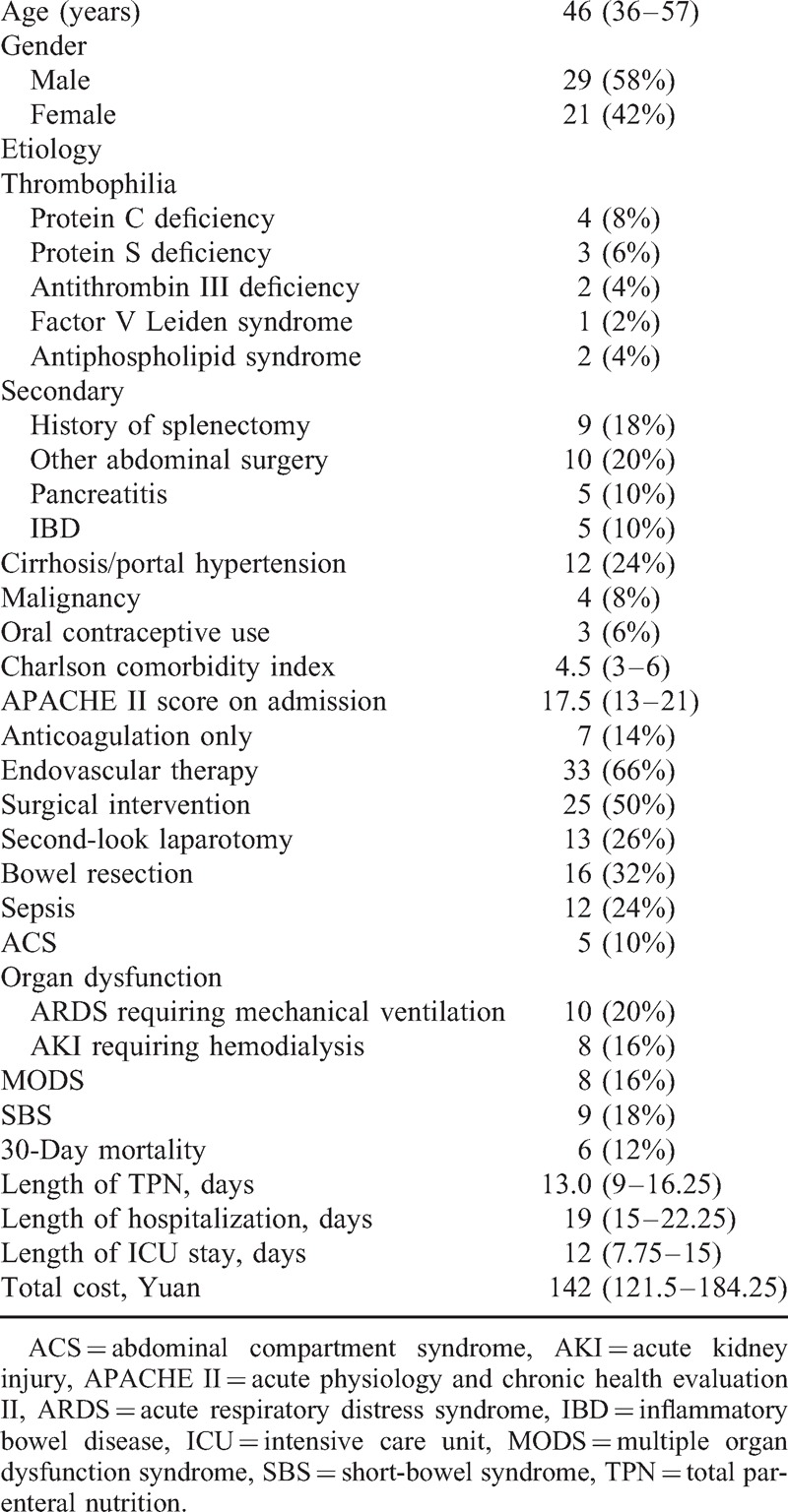
Demographic Data, Etiology, and Clinical Characteristics

Patients received treatments of anticoagulation only (n = 7), endovascular therapy (n = 33), and surgical intervention (n = 25) with bowel resection (n = 16). The overall 30-day mortality was 12%, and hospitalization length was 19 (15–22.25) days. Sepsis developed in 12 patients and ACS in other 5 cases. In intensive care unit (ICU), 10 patients of ARDS received mechanical ventilation, and 8 AKI patients undertook continuous renal replacement therapy. The incidence of MODS and SBS was 16% and 18%, respectively.

### Maximum and Mean D-Dimer Levels and Principal Clinical Variables

**FIGURE 1:**
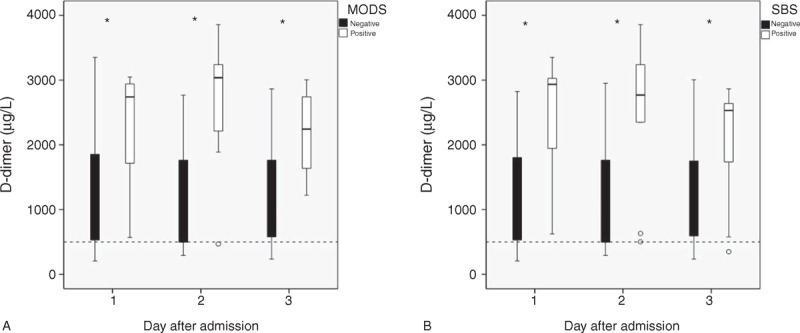
(A) Box plot of the D-dimer level in patients with and without multiple-organ dysfunction syndrome (MODS) on the first 3 days after admission. White boxes, MODS (−); black boxes, MODS (+). (B) Box plot of the D-dimer level in patients with and without short-bowel syndrome (SBS) on the first 3 days after admission. White boxes, SBS (−); black boxes, SBS (+). ∗*P* < 0.05 between patients with and without MODS or SBS; the dashed line means the upper limit of the reference interval.

As shown in Tables 2 and 3, the maximum and mean serum levels of D-dimer in the 3 consecutive days after admission were significantly higher in patients with death within 30 days, surgical intervention, second-look laparotomy, bowel resection, TPN ≥14 days, sepsis, ACS, and ARDS requiring mechanical ventilation, but not AKI requiring hemodialysis (all *P* < 0.05). In addition to the maximum and mean level during the first 3 days, patients with MODS or SBS showed markedly higher D-dimer value in each day (all *P* < 0.05, Tables 2 and 3, Figure 1). No notable disparity of maximum and mean D-dimer value was observed in Tables 2 and 3.

**TABLE 2:**
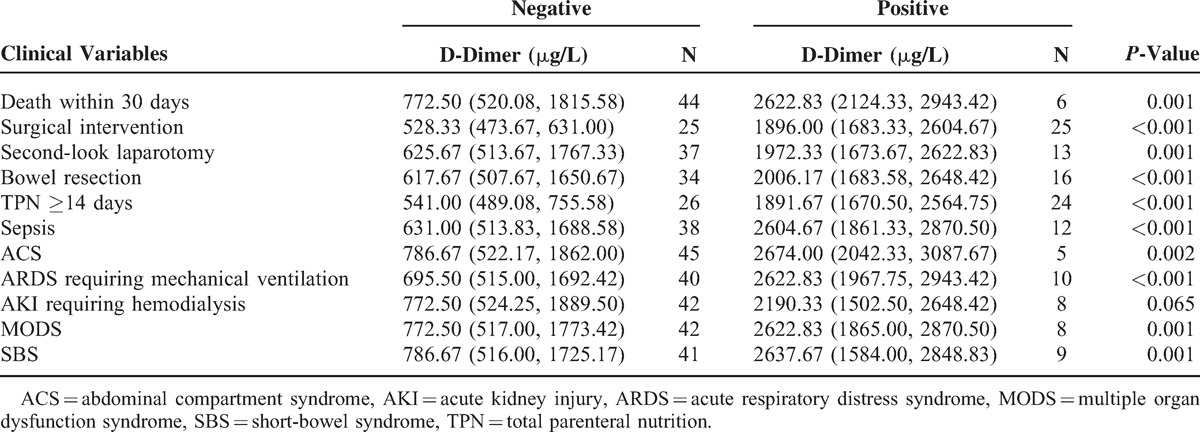
Relation of Maximum Serum D-Dimer Levels and Principal Clinical Variables

**TABLE 3:**
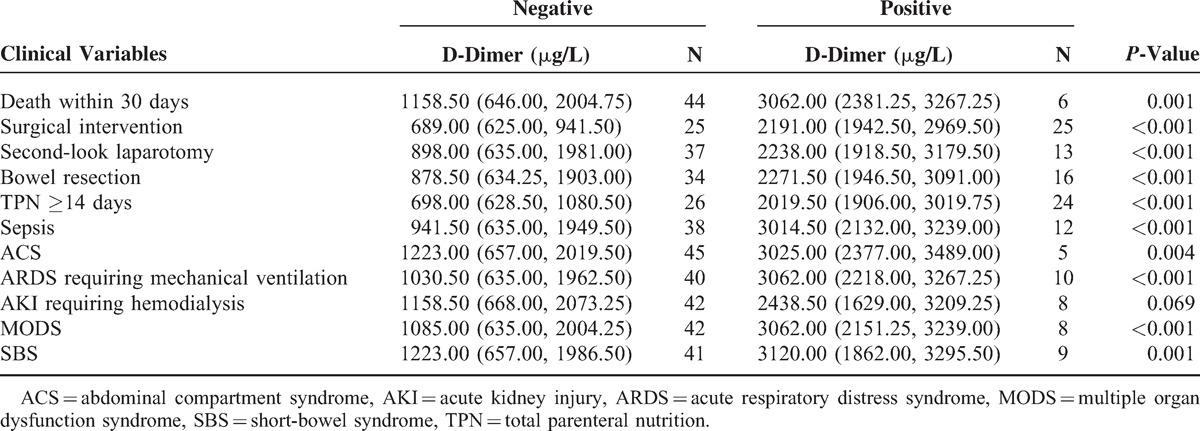
Relation of Mean Serum D-Dimer Levels and Principal Clinical Variables

### Prediction Accuracy of MODS and SBS

Parameters of accuracy for the maximum and mean D-dimer levels, with an optimal cut-off value, in predicting the development of MODS and SBS were listed in Tables 4 and 5. On the ROC curve of maximum and mean D-dimer level for prediction of MODS, the AUC were both 0.87, with an ideal cut-off value of 2447.67 and 2796.00 μg/L (Table 4). In the prediction of SBS, the AUC of maximum and mean D-dimer levels were 0.83 and 0.84, with an ideal cut-off value of 2447.67 and 2796.00 μg/L, respectively (Table 5). To compare each value of AUC, Sn, Sp, +LR, −LR, PP, NP derived from the ROC curve of D-dimer level with those of I-FABP and l-lactate level, both maximum and mean D-dimer levels during the first 3 days of admission showed greater power in prediction of MODS and SBS remarkably (Tables 4 and 5). In the univariate logistic regression analysis, age was not an independent risk factor of SBS and MODS. Both maximum and mean levels of D-dimer, I-FABP, l-lactate were all associated with SBS and MODS, significantly (Tables 6 and 7).

**TABLE 4:**
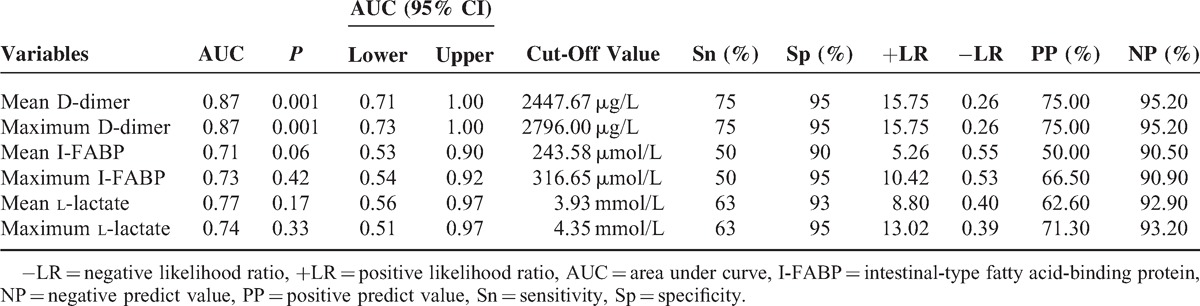
Values of Maximum and Mean Serum D-Dimer, I-FABP, and l-Lactate Level in the Prediction of MODS

**TABLE 5:**
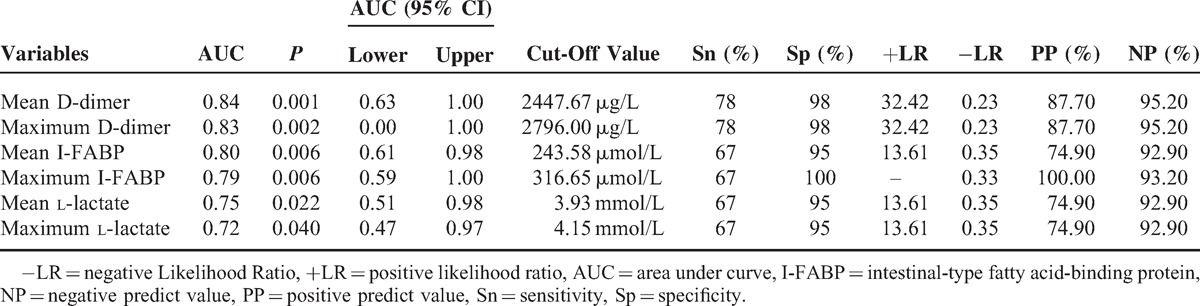
Values of Maximum and Mean Serum D-Dimer, I-FABP, and l-Lactate Level in the Prediction of SBS

**TABLE 6:**
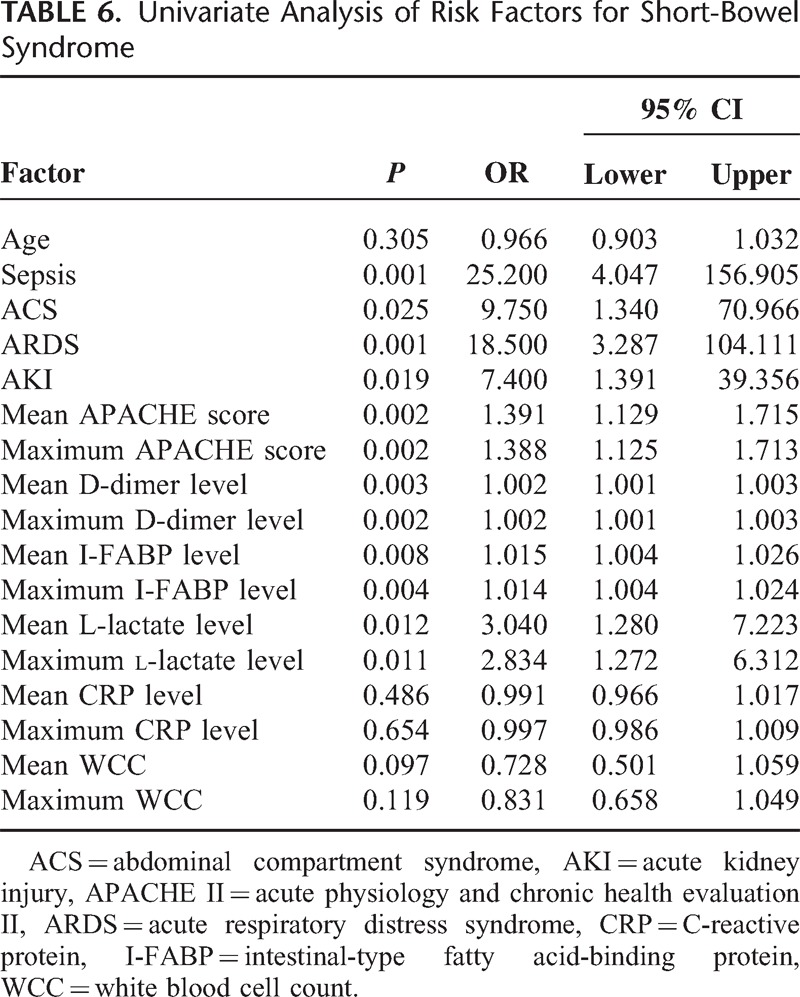
Univariate Analysis of Risk Factors for Short-Bowel Syndrome

**TABLE 7:**
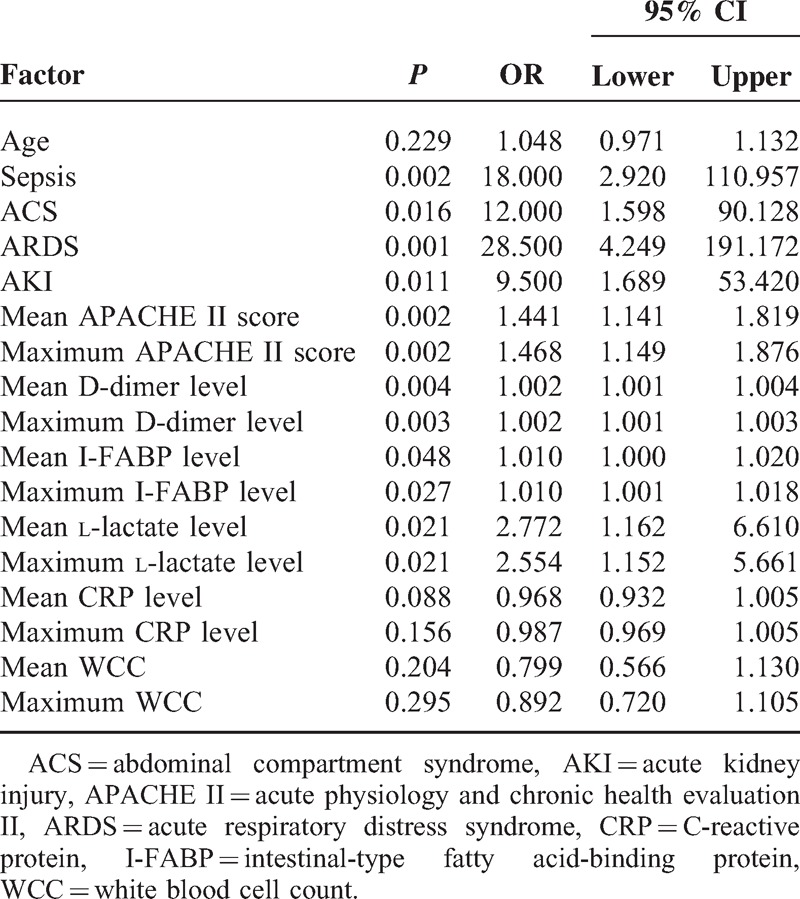
Univariate Analysis of Risk Factors for Multiple Organ Dysfunction Syndrome

### Agreement and Correlation of D-Dimer and Other Significant Parameters

**FIGURE 2:**
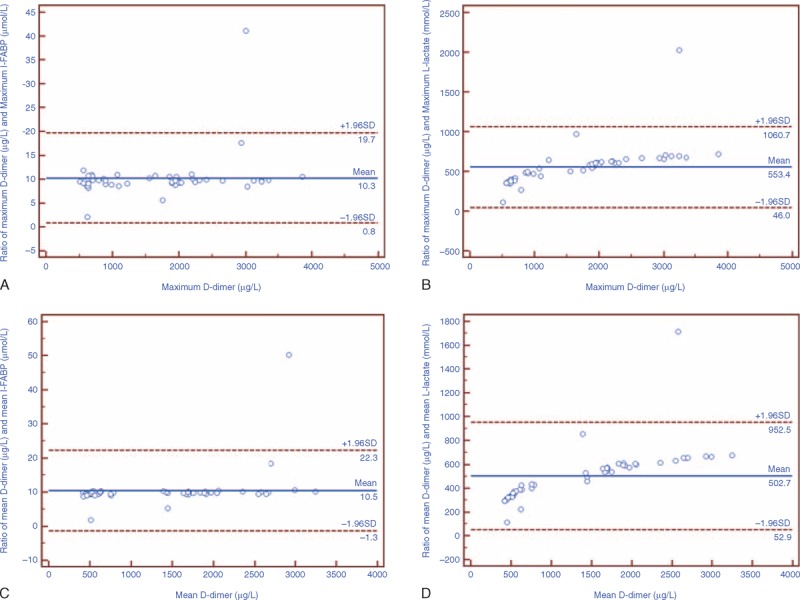
Bland–Altman plot of ratios for serum levels of D-dimer and I-FABP, l-lactate on the first 3 days after admission. The solid line in the middle shows the mean; the upper and lower dashed lines present the 95% confidence interval. (A and B) Well agreement of maximum I-FABP, l-lactate, and D-dimer levels on the first 3 days after admission. (C and D) Well agreement of mean I-FABP, l-lactate, and D-dimer levels on the first 3 days after admission.

**FIGURE 3:**
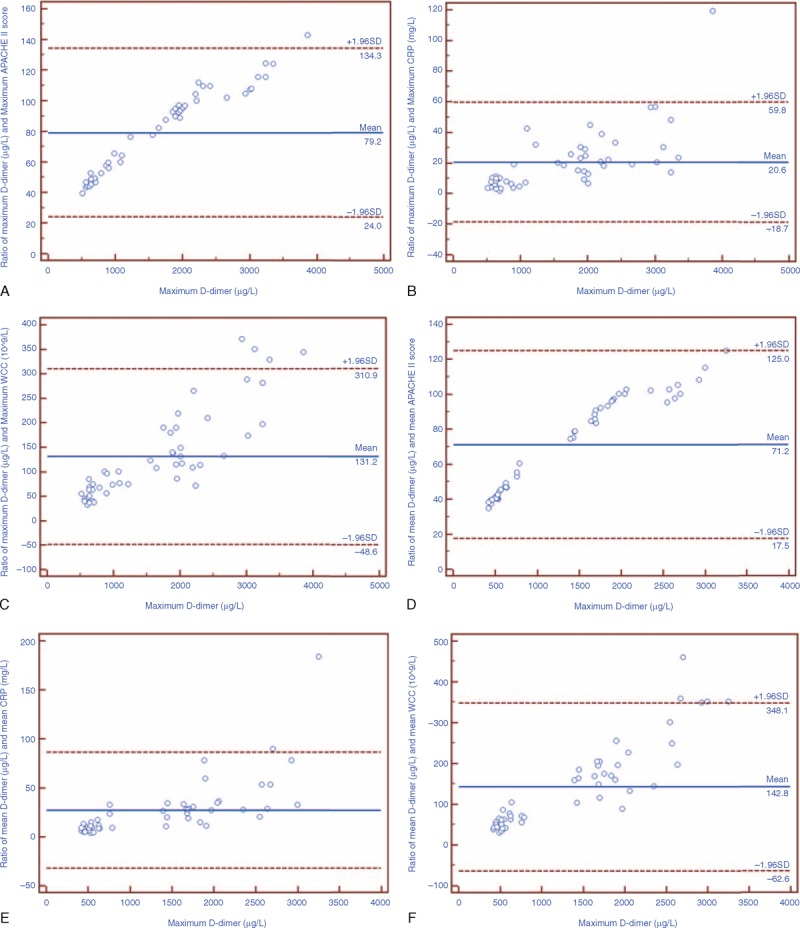
Bland–Altman plot of ratios for serum levels of D-dimer and APACHE II score, CRP level, white blood cell count (WCC). The solid line in the middle shows the mean; the upper and lower dashed lines present the 95% confidence interval. (A–C) Poor agreement of maximum APACHE II score, CRP level, WCC, and serum D-dimer level. (D–F) Poor agreement of mean APACHE II score, CRP level, WCC, and serum D-dimer level.

Figures 2 and 3 show Bland–Altman plots for ratios between the maximum and mean serum D-dimer values and other potential markers. Both the I-FABP and l-lactate were in well agreement with D-dimer while the APACHE II score, CRP level, and WCC were not. For the maximum values, mean ratios between D-dimer and I-FABP, l-lactate were 10.3 (95% CI = 0.8–19.7) and 55.3 (95% CI = 46.0–1060.7). For the mean values, mean ratios between D-dimer and I-FABP, l-lactate were 10.5 (95% CI = −1.3 to 22.3) and 502.7 (95% CI = 52.9–952.5).

**FIGURE 4:**
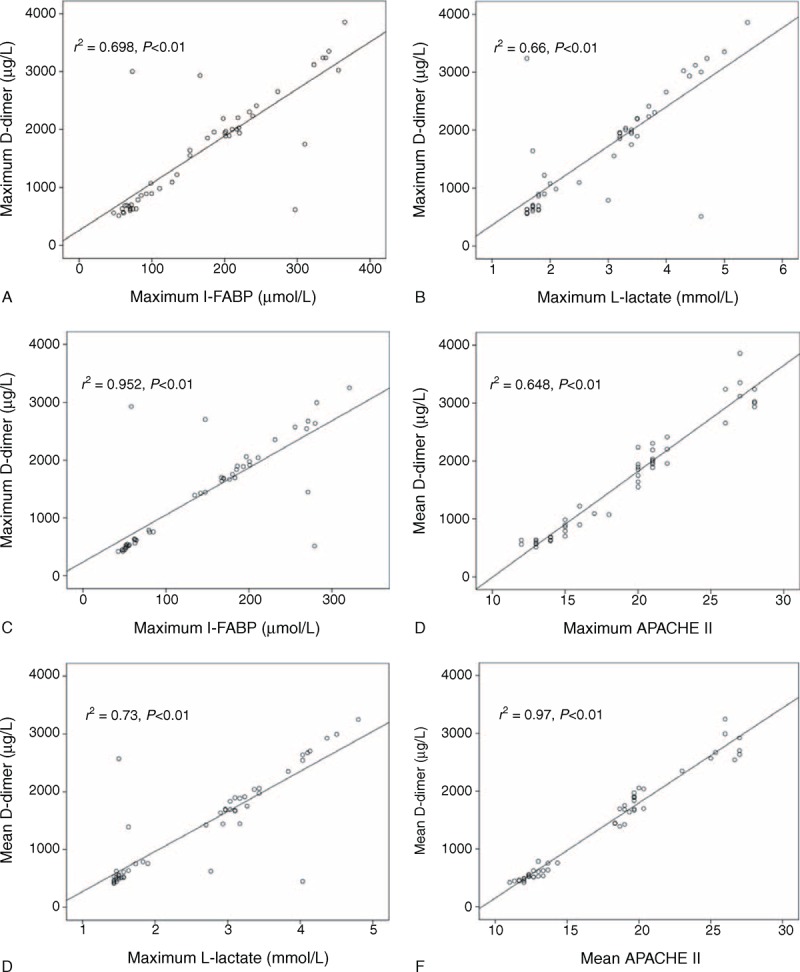
Correlation of serum D-dimer level with intestinal-type fatty acid-binding protein (I-FABP), l-lactate level and acute physiology and chronic health evaluation II (APACHE II) score on the first 3 days after admission. (A–C) Positive correlation of maximum D-dimer level with maximum I-FABP, l-lactate level, and APACHE II score. (D–F) Positive correlation of mean D-dimer level with mean I-FABP, l-lactate level, and APACHE II score. The value of *r*^2^ and *P* were labeled on each figure.

**FIGURE 5:**
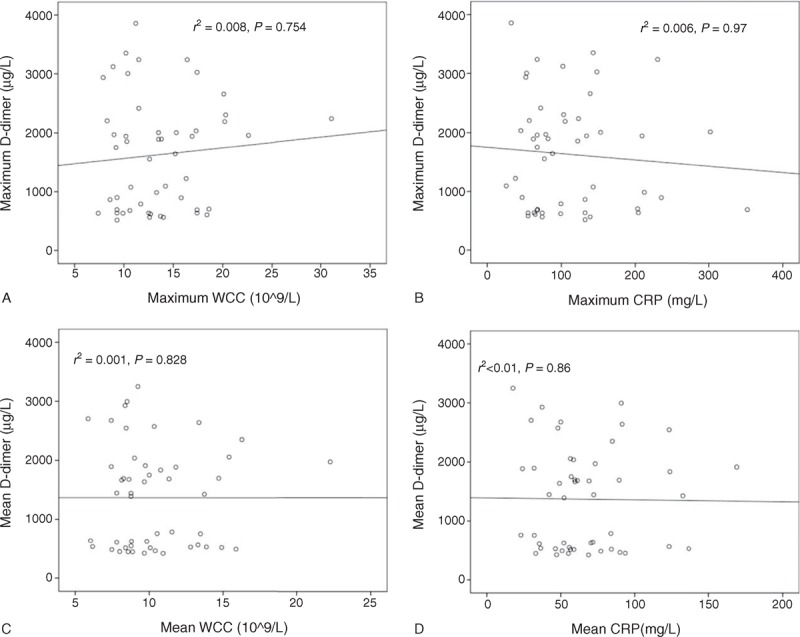
Correlation of serum D-dimer level with white blood cell count (WCC) and C-reactive protein (CRP) level on the first 3 days after admission. (A and B) Negative correlation of maximum D-dimer level with maximum WCC and CRP level. (C and D) Negative correlation of mean D-dimer level with mean WCC and CRP level. The value of *r*^2^ and *P* were labeled on each figure.

Both maximum and mean serum D-dimer levels correlated very well other 2 possible serum severity markers, I-FABP (*r*^2^ = 0.698 & 0.648) and l-lactate (*r*^2^ = 0.66 & 0.73), as well as APACHE II score (*r*^2^ = 0.952 & 0.97) (all P < 0.05, Figure 4). However, there was no linear correlation between D-dimer level and inflammatory markers, WCC, and CRP level (Figure 5). This suggested that D-dimer was an early severity marker of the acute vascular event rather than a non-specific marker of inflammation or necrosis.

## DISCUSSION

Early recognition and timely intestinal revascularization are key factors for decreasing intestinal morbidity and overall mortality of ASMVT. With early diagnosis, it is of great significance to anticipate the disease severity and potential complications during clinical evolution for determining the optimal management of ASMVT. As we know, this is the largest-scale single-center study of ASMVT so far to demonstrate that both the maximum and mean levels of D-dimer in the first 3 days after admission appear to be accurate serum severity markers. ASMVT has a progressive pathophysiology originating from mesenteric venous occlusion and secondary arteriospasm with bowel hypoperfusion. Then activation of the renin–angiotensin–aldosterone pathway leads to extensive splanchnic vasoconstriction.^24^ Regional hypoxia, acidosis, and epithelial injury are associated with massive intestinal bacterial translocation to stimulate systemic inflammatory response syndrome (SIRS).^25,26^ When it progresses to transmural bowel necrosis, sepsis, and MODS would happen. ASMVT carries a high mortality with MODS and extensive intestinal infarction, marked by SBS and permanent intestinal failure requiring long-term total parenteral nutrition or intestinal transplantation.^6,27^ Early evaluation in high-risk patients and resection of necrosed intestinal segments as soon as possible prior to sepsis may reduce the hospital mortality rate.^28^ The measurement of serum D-dimer level is easy, reproducible, and widely used in clinical laboratory. In this context, the predictive value of D-dimer level for MODS and SBS is of extreme interest, as the use of this parameter may enable the decision of prompt surgery and aggressive intensive care measures.

Serum D-dimer level at admission has been extensively reported as a sensitive diagnostic marker of AMI.^11–13,29,30^ But correlation between serum D-dimer levels and disease severity of AMI was failed to found.^14^ The present investigation is the first study to measure the serum level of D-dimer for 3 consecutive days and use both the maximum and mean values, instead of the levels on admission, as predictors for disease evolution of ASMVT. The mechanism underlying the dramatically elevated D-dimer levels in more severe cases could be complex. Extensive activation of fibrinolysis secondary to diffuse formation of microthrombi within intramural venules, vasa recta, and venous arcades, which is associated with serious microcirculatory compromise and greater risk of bowel infarction, could be the dominant cause.^31^ Intestine is the central role of SIRS and a motor of MODS.^32^ Severe coagulative disorder within the intestinal tissue could lead to earlier and larger bowel necrosis, increase the intensity of inflammatory reaction, and contribute greatly to MODS development.

The maximum and mean D-dimer levels, as potential prognostic markers rather than non-specific inflammatory parameters, have consistently shown notably high negative predict value, 95.2% for both mean and maximum value in this study, for AMI in various study populations.^12–14,18,33^ Serum l-lactate and I-FABP levels are the other 2 promising diagnostic markers of ASMVT.^34–36^ The maximum and mean D-dimer values demonstrate more accuracy in prediction of MODS and SBS than l-lactate and I-FABP. Furthermore, both maximum and mean values of D-dimer correlate well with APACHE II score, which is an extensively used risk assessment method for critically ill patients in ICU. Recently, several novel serum makers for AMI (eg, red cell distribution width, mean platelet volume, ischemia-modified albumin, signal peptide-CUB-EGF domain-containing protein 1) are proposed.^37–40^ Their potential as new prognostic marker of ASMVT warrants more research.

In this study, optimum cut-off points for all potential markers were determined by the maximum sum of sensitivity and specificity, instead of maximum sensitivity at the expense of specificity or vice versa. Since ASMVT is a critical illness of high morbidity and mortality that would steeply increase with delayed treatment, a severity marker of high sensitivity is desirable. However, decrease in specificity leads to excess morbidity due to unnecessary surgical procedures. Taking theses factors into account, best cut-off points represented by the maximum sum of sensitivity and specificity are considered to predict incidence of MODS and SBS. Remarkably high serum levels of D-dimer at the moment of diagnosis may justify an early laparotomy, whereas significantly low levels could be the reason for a conservative policy of systemic anticoagulation and intensive care. Furthermore, these laboratory parameters should be used as a supplement to disease history, physical examination, and radiology evaluation. This is especially true for levels closely approaching the cut-off point. Owing to the large colinearity, combined use of the described markers unlikely yielded better prediction accuracy.

Of note, the existence of several commercially available D-dimer assays with different calibrators may result in variability of their performance characteristics.^41^ Thus, the D-dimer cut-off points in our study cannot automatically be generalized to other hospital settings with a different D-dimer assay.^42^ Moreover, D-dimer is not the only raised parameter of fibrinolysis in patients with mesenteric venous occlusion. In contrast to other parameters, D-dimer is a stable molecule of sufficiently long half-life (4–8 h) to attain high serum levels when the generation is accelerated.^43^ Nevertheless, the D-dimer test seems less suitable in non-acute phase (≥1 week) of ASMVT.^9,44^

At present, there are great challenges in management of ASMVT with wide international practice variation. These challenges are largely reflective of the absence of level IA evidence to guide routine clinical practice. D-dimer level as a part of clinical decision rules is a promising tool to improve cost-effective clinical care. It assists the clinician to judiciously use invasive evaluation, select the right treatment setting (ICU or ward) for initial therapeutic management (emergent laparotomy, endovascular therapy, or systemic anticoagulation) and predict, which patients will derive net benefit from anticoagulant therapies only. However, knowledge translation barriers and wide international practice variation warrant future researches.

There are some certain limitations in this study. First of all, due to the rareness of ASMVT, we had to conduct an observational study without control groups, and choose a non-parametric test with potential low statistical power and uncertainty. However, a single-center large-series study of ASMVT is very difficult to obtain in a prospective way. Moreover, it is important to acknowledge that the great statistical significance in this study may be attributed to that D-dimer levels of many patients were very low on the first 3 days of admission. Additionally, as some severe cases presented sepsis or MODS during the first 3 days after admission, the prognostic value of repeated measurements of D-dimer were less powerful in such cases. Another limitation pertains to that some patients receive immediate anticoagulation at emergency room, and D-dimer values could be lowered by therapeutic doses of warfarin and heparin.^45^ Therefore, the D-dimer values should be used flexibly and legitimately, with a combination of serum level on admission and consecutive values in clinical practice.

In conclusion, it is demonstrated in this study that D-dimer level could be an early, easy, and effective serum marker correlating well with the clinical evolution and outcome of ASMVT, although this observation requires further verification. D-dimer level is a specific parameter of high predictive power and ability in assisting decision-making of proper and timely treatment of ASMVT. A large multi-center prospective study is warranted to confirm these results further.
